# Network meta-analysis of survival data with fractional polynomials

**DOI:** 10.1186/1471-2288-11-61

**Published:** 2011-05-06

**Authors:** Jeroen P Jansen

**Affiliations:** 1 Mapi Values, Boston, USA

## Abstract

**Background:**

Pairwise meta-analysis, indirect treatment comparisons and network meta-analysis for aggregate level survival data are often based on the reported hazard ratio, which relies on the proportional hazards assumption. This assumption is implausible when hazard functions intersect, and can have a huge impact on decisions based on comparisons of expected survival, such as cost-effectiveness analysis.

**Methods:**

As an alternative to network meta-analysis of survival data in which the treatment effect is represented by the constant hazard ratio, a multi-dimensional treatment effect approach is presented. With fractional polynomials the hazard functions of interventions compared in a randomized controlled trial are modeled, and the difference between the parameters of these fractional polynomials within a trial are synthesized (and indirectly compared) across studies.

**Results:**

The proposed models are illustrated with an analysis of survival data in non-small-cell lung cancer. Fixed and random effects first and second order fractional polynomials were evaluated.

**Conclusion:**

(Network) meta-analysis of survival data with models where the treatment effect is represented with several parameters using fractional polynomials can be more closely fitted to the available data than meta-analysis based on the constant hazard ratio.

## Background

Healthcare decision-making requires comparisons of all relevant competing interventions. If the available evidence consists of a network of multiple randomized controlled trials (RCTs) involving treatments compared directly or indirectly or both, it can be synthesized by means of network meta-analysis [1-4]. Network meta-analysis of survival data is often based on the reported hazard ratio, which relies on the proportional hazards assumption.

The proportional hazards assumption that underlies current approaches of evidence synthesis of survival outcomes is not only often implausible, but can have a huge impact on decisions based on cost-effectiveness analysis. In extreme cases survival curves intersect and the hazard ratio is not constant. Furthermore, even if survival functions do not intersect, the hazard functions might and the assumption is violated. For cost-effectiveness evaluations of competing interventions that aim to improve survival, differences in expected survival between the competing interventions are of interest. Common practice is to assume a certain parametric survival function for the baseline intervention (e.g. Weibull) and apply the treatment specific constant hazard ratio obtained with the (network) meta-analysis to calculate a corresponding survival function enabling comparisons of expected survival. Since the tail of the survival function has a great impact on the expected survival, violations of the constant hazard ratio can lead to severely biased estimates. Hence, the proportional hazards assumption has become a source of concern in drug reimbursement based on cost-effectiveness evidence.

As an alternative to a network meta-analysis of survival data in which the treatment effect is represented by a single parameter, i.e. the hazard ratio, a multi-dimensional treatment effect approach is presented. With fractional polynomials the hazard over time is modeled by which the treatment effect is represented with multiple parameters [5]. With this approach a network meta-analysis of survival can be performed with models that can be fitted more closely to the data. With these parametric hazard functions, expected survival can be calculated to facilitate cost-effectiveness analysis. The method is illustrated with an example.

## Methods

### Fractional polynomials and the hazard function

Royston and Altman introduced fractional polynomials as an extension of polynomial models for determining the functional form of a continuous predictor [5]. These models are well suited for nonlinear data. In contrast to categorizing continuous predictors, the analysis is no longer dependent on the number and choice of cut points [6]. Fractional polynomials have been used in many applications including survival and meta-regression analysis [7-9].

By transforming *t*, a continuous variable, in a linear model the first-order fractional polynomial model is obtained:(1)

The power *p *is chosen from the following set: -2. -1, -0.5, 0, 0.5, 1, 2, 3 with *t*^0 ^= log *t*

The second order fractional polynomial is defined as:(2)

If *p_1 _= p_2 _= p *the model becomes a 'repeated powers' model:(3)

Royston and Altman showed that by varying *p_1 _*and *p_2 _*and the parameters *β*_0, _*β*_1 _and *β*_2 _a wide range of curve shapes can be obtained [5,6,8,10,11].

The first order fractional polynomial for the hazard at time *t *of a two arm treatment B versus A randomized controlled trial can be presented as follows:(4)

where: *h_kt _*reflect the hazard with treatment *k *at time *t*. The vector  reflects the parameters *β*_0 _and *β*_1 _of the 'baseline' treatment A, whereas the vector  reflects the difference in *β*_0 _and *β*_1 _of the log hazard curve for treatment B relative to A. The parameter *d_0 _*corresponds to the treatment effect with a proportional hazard model. Under the proportional hazards assumption *d_1 _*equals 0. If *d_1 _*≠ 0, *d*_1 _reflects the change in the log hazard ratio over time. Hence, by incorporating *d_1 _*in addition to *d*_0 _a multi-dimensional relative treatment effect is used rather than single parameter for the relative treatment effect.

Hazard functions can have different shapes, including a constant hazard over time, a linear increasing or decreasing hazard over time, and bathtub shaped. If in equation 4 *β*_1 _equals 0, a constant log hazard function is obtained, reflecting exponentially distributed survival times. If *β*_1 _≠ 0 and *p *= 1 a linear hazard function is obtained which corresponds to a Gompertz survival function. If *β*_1 _≠ 0 and *p *= 0 a Weibull hazard function is obtained, and  reflects the difference in respectively the scale and shape of the Weibull log hazard curve for treatment B relative to A. Extending the first-order fractional polynomial hazard function to a second-order fractional polynomial increases the possible (differences in) shapes even further. Hence, modeling the hazard function of competing interventions with fractional polynomials provides a general framework that includes some of the commonly used parametric survival functions and does not rely on the constant hazard ratio assumption.

### Network meta-analysis model for survival data using fractional polynomials

Network meta-analysis has been presented as an extension of traditional meta-analysis by including multiple different pairwise comparisons across a range of different interventions. Meta-analysis models for the comparison of treatment B versus A can be extended to models allowing simultaneous comparisons of B versus A as well as C versus A [1-4]. To appreciate the randomization of the different studies in the evidence synthesis, a study of a certain pairwise comparison has to be 'linked' to any of the other studies in the network. When the network consists of AB-trials, AC-trials, as well as BC trials, we have a mixture of direct and indirect comparisons and these analyses have been called mixed treatment comparisons (MTC) [3].

For a network meta-analysis, the similarity and consistency relation needs to hold regarding the estimated model parameters [3,12,13]. If AB trials and AC trials are comparable on effect modifiers (i.e. covariates that affect the relative treatment effect), then an indirect estimate for the relative effect of C versus B (*d_BC_*) can be obtained from the estimates of the effect of B versus A (*d_AB_*) and the effect of C versus A (*d_AC_*): *d_BC _= d_AC _- d_AB_*. In essence, this implies that the same *d_BC _*is obtained as would have been estimated in a three arm randomized ABC trial. In general, for a model described by the function *f_x_*(*t*) where x = A, B, or C, we have: (*f_C_*(*t*)- *f_B_*(*t*)) = (*f_C_*(*t*)- *f_A_*(*t*))-(*f_B_*(*t*)- *f_A_*(*t*)). For a network meta-analysis of survival data, the comparison can be performed on the log hazard ratio, and this relation needs to apply to every timepoint *t*: ln(*HR_BC_*(*t*))- ln(*HR_AC_*(*t*))- ln(*HR_AB_*(*t*)) with *HR_BC_*(t) reflecting the hazard ratio of C relative to B at time *t*. Based on equation 4 it follows that:(5)

Hence, the differences in the model parameters *β*_0 _and *β*_1 _of the first order fractional polynomials are independent of time. Furthermore, according to equation 5 the difference in *β*_0 _and *β*_1 _of the BC comparison can be described by the difference in these parameters for the AC comparison and AB comparison. Given this relation, a network meta-analysis can be performed based on the differences in *β*_0 _and *β*_1 _of log hazard curves across studies. Similarly, the transitivity assumption holds for fractional polynomials of any order.

Using a similar notation as Cooper et al. [13], the random effects model for a network meta-analysis of survival data based on a fractional polynomial of order M for *k *treatments labeled A, B, C, etc can be described as:(6)

where *h_jkt _*reflects the underlying hazard rate in trial *j *for intervention *k *at time point *t*. The vectors  are trial-specific and reflect the parameters *β*_0_, *β*_1_,..., *β_M _*of the comparator treatment, whereas the vectors  reflect the study specific difference in *β*_0_, *β*_1_,..., *β_M _*of the log hazard curve for treatment *k *relative to comparator treatment *b*. and are drawn from a multivariate normal distribution with the pooled estimates expressed in terms of the overall reference treatment A with . For example, , , etc. Σ is the between study covariance matrix to reflect heterogeneity which is assumed constant for all treatment comparisons where σ*_m _*represent the variance for *δ_mjbk _*(i.e. the difference in *β_m_*) and *ρ*_01_, *ρ*_02_,..., *ρ*_*M*-1,*M*_is the correlation between these parameters. Of key interest from the analyses are the pooled estimates of *d_mAk _*and estimates for the heterogeneity. Please note that the HR is changing over time once *d*_m≥1 _is different from 0.

Under a fixed effects model the multivariate normal distribution with the pooled estimates will be replaced with  and as a result the between study covariance matrix does not need to be estimated. When only for *d*_0*Ak *_heterogeneity is assumed, and the other effect parameters *d*_1*Ak*_,..., *d_MAk _*are fixed, then  is replaced with *δ*_0*jbk*_~*Normal*(*d*_0*Ak*_-*d*_0*Ab*_, *σ*^2^) and .

A random effects model with only a heterogeneity parameter for *d*_0*Ak *_implies that the between study variance of the log hazard ratios remains constant over time. Random effects models with (additional) heterogeneity parameters for *d*_1*Ak*_,..., *d_MAk _*have the flexibility to capture between study variance regarding changes in the log hazard ratios over time.

The random effects fractional polynomial model in equation 6 treats multiple-arm trials (>2 treatments) without taking account of the correlations between the trial-specific δs that they estimate. Bayesian random effects fractional polynomials models with only a heterogeneity parameter for *d*_0*Ak *_can be easily extended to fit trials with 3 or more treatment arms by decomposition of a multivariate normal distribution as a series of conditional univariate distributions [13]. If  then the conditional univariate distributions for arm *i *given the previous *1,....(i-1) *arms are:

Different values for the powers *p_1 _*and *p_2 _*of the fractional polynomials correspond to different models. The best fitting model can be selected based on goodness-of-fit comparisons. The goodness of fit can be computed as the difference between the deviance for the fitted model and the deviance for the saturated model (which fits the data perfectly). Within a frequentist framework the Akaike information criterion (AIC) can be used for model selection [14]. In a Bayesian framework the Bayesian information criterion (BIC) or deviance information criterion (DIC) can be used [15,16].

### Illustrative example

To understand how the analytical approach proposed can be applied in practice, an example is presented for oncology where trials are typically focused on overall (and progression free) survival.

Lung cancer is a leading cause of cancer mortality in both men as well as women, with non-small cell lung carcinoma (NSCLC) accounting for 80% of all cases [17]. Second line treatment for advanced NSCLC includes docetaxel and pemetrexed [18]. Gefitinib has been studied as second line treatment as well.

**Figure 1:**
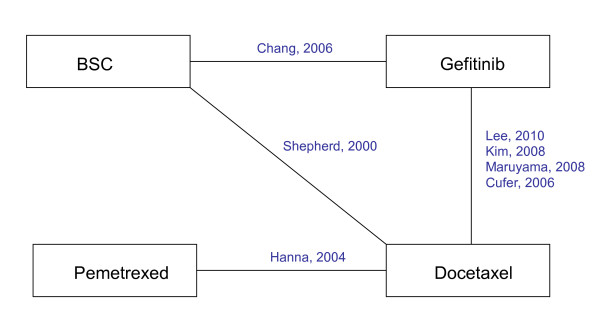
**Network of randomized controlled trials**.

**Figure 2:**
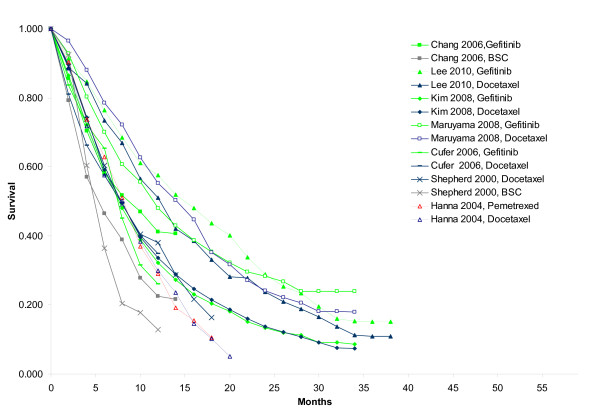
**Survival as observed in individual studies**.

A literature search identified seven RCTs comparing docetaxel with best-supportive care (1 study), gefitinib with best-supportive care (1 study), docetaxel with gefitinib (4 studies), and docetaxel with pemetrexed (1 study) [19-25]. The network of RCTs is presented in Figure 1 and shows that for the comparisons of BSC, docetaxel and gefitinib both direct and indirect evidence is available. For each treatment arm in each study reported Kaplan-Meier curves were digitized (Engauge Digitaliser v4.1) In Figure 2 the scanned survival proportions are presented. This aggregate data was analyzed with fractional polynomial network meta-analysis models.

Whilst network meta-analysis can be performed with a frequentist or a Bayesian approach, for this manuscript the focus is on the Bayesian approach. Within the Bayesian framework, analyses consist of data, likelihood, parameters, a model, and prior distributions. More specifically, Bayesian analysis involves the formal combination of a prior probability distribution that reflects a prior belief of the possible values of the parameters of the model with a likelihood distribution of the model parameters based on the observed data in the different studies to obtain a posterior probability distribution of these [26-28].

The scanned survival curves can be divided into multiple consecutive intervals over the follow-up period. Extracted survival proportions were used to calculate the incident number of deaths for each interval and patients at risk at the beginning of that interval. A binomial likelihood distribution of the incident number of deaths for every interval [*t,t+Δt*] (Δ*t *is the time from *t *to *t*+1) of the Kaplan-Meier curves can be described according to:(7)

Where *r_jkt _*is the observed number of incident deaths in the interval [*t,t+Δt*] for study *j *and treatment *k*. *n_jkt _*Is the number of subjects alive at *t*, adjusted for the subjects censored in the interval [*t,t+Δt*]. *p_jkt _*is the observed cumulative incidence of deaths in the interval [*t,t+Δt*]. In the appendix more detail is provided how a dataset for *n_jkt _*and *r_jkt _*can be obtained from the Kaplan-Meier curve taking into account censoring in the interval [*t,t+Δt*]. In Table 1 the incident deaths and patients at risk for every 2-month period of the individual studies are presented. When the time interval is relatively short, the hazard rate can be assumed constant within the time interval, and the hazard rate *h_jkt _*is:(8)

**Table 1:** Number of deaths and patients at risk for every consecutive 2-month period as extracted from digitized survival curves (see appendix for details)

Study 1				Study 2				Study 3				Study 4				Study 5				Study 6				Study 7			
Lee et al., 2010				Chang et al. 2006				Kim et al., 2008				Maruyama et al., 2008				Hanna et al., 2004				Cufer et al., 2006				Shepherd et al., 2000			
**Gefitinib**		**Docetaxel**		**BSC**		**Gefitinib**		**Gefitinib**		**Docetaxel**		**Gefitinib**		**Docetaxel**		**Pemtrexed **		**Docetaxel**		**Gefitinib**		**Docetaxel**		**BSC**		**Docetaxel**	

**r**	**n**	**r**	**n**	**r**	**N**	**r**	**n**	**r**	**n**	**r**	**n**	**r**	**n**	**r**	**n**	**r**	**n**	**r**	**n**	**r**	**n**	**r**	**n**	**r**	**n**	**r**	**n**

7	81	9	76	22	107	34	235	98	723	73	710	18	245	9	244	27	283	33	288	11	68	14	73	17	222	49	441
5	74	4	67	24	85	35	201	104	625	109	637	30	226	21	233	47	256	48	255	8	57	11	59	71	205	67	392
7	69	8	64	11	61	29	166	94	518	102	503	25	197	23	214	31	209	41	207	5	49	6	48	53	134	60	325
6	62	5	56	8	50	15	137	79	424	66	401	22	169	15	189	33	178	21	166	14	44	6	42	70	160	63	340
6	55	8	51	12	42	11	122	64	336	68	339	12	148	23	173	40	144	34	144	9	31	7	36	12	90	49	277
3	49	4	38	6	30	14	110	46	272	41	271	18	127	17	140	17	78	17	78	4	21	4	29	22	78	14	228
4	46	6	34	1	24	1	97	35	225	32	228	10	98	9	105	21	61	13	61	8	18					55	221
3	42	2	28					30	190	29	196	8	77	10	87	8	40	18	48							41	166
3	38	4	26					14	131	18	139	6	63	15	69	11	33	9	30							30	125
3	35	3	22					14	117	16	121	4	47	4	44			10	21								
5	29	3	18					14	83	13	89	3	35	5	35												
4	24	2	15					8	69	10	76	1	29	3	25												
3	21	1	13					5	50	6	46	1	25	2	18												
1	18	1	12					3	45	5	40	2	18	1	14												
3	17	2	10					6	31	4	24			1	10												
3	14	2	8							3	20																
1	12																										

In this example fixed and random effects first and second order fractional polynomial models were used with powers chosen from the following set: -2. -1, -0.5, 0, 0.5, 1, 2, 3 with *t*^0 ^= log *t *according to eq. 6. Two different random effects second order fractional polynomial models were compared: one model with a heterogeneity parameter for *d_0_*, and one model with heterogeneity parameters for all three treatment parameters *(d_0_, d_1 _*or *d*_2_). Although random effects models with a heterogeneity parameter for only *d_1 _*or *d*_2 _can be estimated as well, these were considered less appropriate because these models assume that heterogeneity in treatment effects only develop over time, and is not present at treatment initiation. In other words: heterogeneity is only a function of time, and not (also) a function of differences in patient characteristics across studies. If only one heterogeneity parameter is (to be) used, it should be for *d_0 _*because it assumes constant variance for the complete follow-up period.

The non-informative prior distributions as used for the parameters of the random effects second-order fractional polynomial model with heterogeneity corresponding to *d_0_, d_1 _*and *d*_2 _are presented (according to equation 6):(9)

For a first order fractional polynomial model these 3-dimensional multivariate prior distributions are reduced to bivariate normal distributions. With a random effects model, where only for *d_0 _*a heterogeneity parameter is used, the corresponding prior distribution can be defined as σ ~ *uniform*(0,2). When all relative effects parameters are assumed fixed, there is no heterogeneity to be estimated, and no such prior distribution needs to be defined.

The parameters of the different models were estimated using a Markov Chain Monte Carlo (MCMC) method as implemented in the WinBUGS software package [29]. (See appendix for the code.) The WinBUGs sampler, using two chains, was run for 30000 iterations for the models and these were discarded as 'burn-in' and the model was run for a further 50 000 iterations on which inferences were based. Convergence of the chains was confirmed by the Gelman-Rubin statistic.

The DIC was used to compare the goodness-of-fit of different fixed and random effects models with first and second order fractional polynomials with different powers. DIC provides a measure of model fit that penalizes model complexity according to [16].  is the posterior mean residual deviance [15], *pD *is the 'effective number of parameters' and  is the deviance evaluated at the posterior mean of the model parameters. The model with the lowest DIC, is the model providing the 'best' fit to the data. For every combination of *p1 *and *p2 *the DIC was determined. The powers *p1 *and *p2 *corresponding to the best fitted fixed effects models were also used to evaluate corresponding random effects models.

## Results

### Illustrative example

The model fit statistics for the different models are presented in Table 2. The fixed effects Weibull model (*p1 *= 0) was one of the worst regarding goodness-of-fit. Of the first order fractional polynomial models, the model with power *p1 *= -2 was the best fit. Adding a second time- related effect to this first order fractional polynomial model dramatically improved the model fit. Although the model with *p1 *= -2 and *p2 *= 1 has the lowest DIC of all the fixed effects models evaluated, the model with *p1 *= -2 and *p2 *= 2 and the model with *p1 *= -2 and *p2 *= 3 deserve consideration as well because these are within 1-2 points of the "best" model [16]. However, the modeled hazard function with *p2 *= 1 is not as sensitive to small sample fluctuations near the end of the follow-up of each study as the models with *p2 *= 2 *or p2 *= 3. To facilitate the extrapolation of the survival curves beyond the trial period, the model with *p1*= -2 and *p2 *= 1 was considered the most appropriate fixed effects model. The corresponding random effects models showed similar values for the DIC, and as such the random effects models were considered more appropriate. The model with a heterogeneity parameter for *d_0 _*only showed more stable parameter estimates than the random effects model with heterogeneity parameters for *d_0_, d_1 _*and *d*_2_. Given the similar fit of these random effect models, the model with one heterogeneity parameter was used.

**Table 2:** Goodness-of-fit estimates for fixed and random effects fractional polynomial models for different powers p1 and p2.

Power *p1*	Power *p2*	Dbar	Dhat	pD	DIC
**Fixed effects models**					
-2	-	904.5	884.6	19.9	924.4
-1	-	916.2	896.3	19.8	936.0
-0.5	-	925.5	905.6	19.9	945.3
0^a^	-	934.6	915.2	19.3	953.9
0.5	-	942.8	923.5	19.4	962.2
1^b^	-	948.7	929.2	19.4	968.1
2	-	957.3	937.5	19.9	977.2
3	-	964.5	944.3	20.2	984.7

-2	-2	865.5	835.8	29.8	895.3
-2	-1	857.2	827.0	30.2	887.4
-2	-0.5	850.9	821.2	29.7	880.6
-2	0	844.4	815.2	29.1	873.5
-2	0.5	840.2	810.6	29.7	869.9
***-2***	***1***	***837.1***	***807.1***	***30.1***	***867.2***
-2	2	837.3	807.2	30.1	867.4
-2	3	837.8	808.2	29.6	867.4

-1	-1	848.4	819.8	28.6	877.0
-1	-0.5	849.0	820.1	28.9	877.9
-1	0	840.9	812.6	28.3	869.2
-1	0.5	840.2	811.4	28.8	869.0
-1	1	839.8	809.8	30.0	869.8
-1	2	844.3	814.0	30.2	874.5
-1	3	850.4	820.4	30.0	880.5

-0.5	-0.5	840.5	811.6	28.9	869.4
-0.5	0	842.2	813.6	28.6	870.8
-0.5	0.5	842.4	812.3	30.1	872.5
-0.5	1	843.6	813.9	29.7	873.3
-0.5	2	851.9	822.9	29.1	881.0
-0.5	3	861.4	831.4	30.0	891.5

0	0	845.8	817.2	28.6	874.4
0	0.5	849.8	821.6	28.2	878.0
0	1	854.6	823.4	31.2	885.8
0	2	862.5	833.3	29.1	891.6
0	3	874.2	844.6	29.6	903.9

0.5	0.5	854.3	830.6	23.8	878.1
0.5	1	860.0	831.8	28.2	888.1
0.5	2	876.4	846.7	29.7	906.1
0.5	3	888.3	858.8	29.4	917.7

1	1	871.4	842.0	29.4	900.8
1	2	887.2	858.3	28.9	916.2
1	3	902.3	871.7	30.6	932.9

2	2	907.8	880.0	27.9	935.7
2	3	921.2	892.2	29.0	950.2

3	3	934.6	906.9	27.7	962.4

**Random effects models**					
-2	-	904.4	883.2	21.3	925.7
0^a^	-	934.4	911.4	23.0	957.4
-2^c^	1	835.9	803.9	32.0	867.9
***-2^d^***	***1***	***836.1***	***805.1***	***31.0***	***867.1***

^a ^Corresponding to Weibull distribution for hazard over time; ^b ^Corresponding to Gompertz distribution for hazard over time; ^c ^Random effects model with heterogeneity for all three effect parameters; ^d ^Random effects model with heterogeneity for d_0_

**Figure 3:**
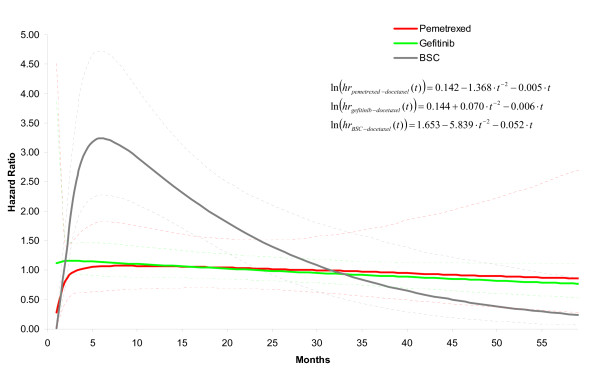
**Hazard ratio over time for each of the interventions relative to docetaxel as obtained with random effects second order fractional polynomial (p1 = -2, p2 = 1) network meta-analysis model**. (Corresponding parameter estimates are presented in Table 3: *d_0Ak_*, *d_1Ak_*, *d_2Ak_*)

Table 3 provides parameter estimates for the fixed effects first and second order fractional polynomial models with *p1 *= -2 and *p2 *= 1, as well as the corresponding random effects model with a heterogeneity parameter for *d_0 _*. Based on the pooled relative treatment effects regarding *β*_0_, *β*_1 _and *β*_2 _of each intervention relative to docetaxel (*d*_0*Ak*_, *d*_1*Ak*_, and *d*_2*Ak *_with k = B,C,D corresponding to respectively gefitinib, BSC, and pemetrexed) the corresponding hazards ratios as a function of time were obtained: ln(*HR_Ak_*) = *d_0Ak _*+ *d*_1*Ak *_· *t*^-2 ^+ *d*_2*Ak *_· *t*. The hazard ratios over time obtained with the random effects model are presented in Figure 3. It is obvious that the assumption of constant hazards ratio does not apply to any comparison with BSC involved. Although for the comparison of gefitinib relative to docetaxel a constant hazard ratio over time might be defended, the additional indirect evidence via BSC for this comparison clearly does not allow this assumption. Based on this observation, one can argue that *d_1 _*and *d*_2 _for gefitinib and pemetrexed relative to docetaxel can be set to zero, and that *d_1 _*and *d*_2 _only need to be estimated for BSC versus docetaxel. However, it has to be realized that by making that assumption the uncertainty regarding the proportional hazards assumption for gefitinib and pemetrexed is no longer taken into consideration.

**Table 3:** Model parameter estimates for different fractional polynomial network meta-analysis models.

		Fixed effects model, first order fractional polynomial		Fixed effects model, second order fractional polynomial		Random effects model (d_0_), second order fractional polynomial	
		**Median of posterior distribution**	**95% Credible Interval**	**Median of posterior distribution**	**95% Credible Interval**	**Median of posterior distribution**	**95% Credible Interval**

*p1*	power 1	-2		-2		-2	
*p2*	power 2			1		1	

Pooled estimate for difference in *β_0 _*							
*d_0AB_*	gefitinib vs. docetaxel	-0.003	(-0.111; 0.112)	0.158	(-0.065; 0.409)	0.144	(-0.156; 0.411)
*d_0AC_*	BSC vs. docetaxel	0.770	(0.598; 0.939)	1.674	(1.108; 2.193)	1.653	(1.039; 2.167)
*d_0AD_*	pemetrexed vs. docetaxel	0.020	(-0.189; 0.243)	0.141	(-0.483; 0.744)	0.142	(-0.540; 0.761)

Pooled estimate for difference in *β_1_*							
*d_1AB_*	gefitinib vs. docetaxel	0.634	(-0.429; 1.726)	-0.003	(-1.470; 1.362)	0.070	(-1.330; 1.542)
*d_1AC_*	BSC vs. docetaxel	-2.507	(-4.281; -0.783)	-5.858	(-8.503; -3.228)	-5.839	(-8.265; -3.131)
*d_1AD_*	pemetrexed vs. docetaxel	-0.995	(-3.344; 1.381)	-1.359	(-4.604; 1.885)	-1.368	(-4.511; 1.814)

Pooled estimate for difference in *β_2_*							
*d_2AB_*	gefitinib vs. docetaxel			-0.007	(-0.016; 0.002)	-0.006	(-0.015; 0.003)
*d_2AC_*	BSC vs. docetaxel			-0.053	(-0.082; -0.023)	-0.052	(-0.079; -0.023)
*d_2AD_*	pemetrexed vs. docetaxel			-0.005	(-0.032; 0.023)	-0.005	(-0.031; 0.022)
*σ_d0_*	Standard deviation of *d_0 _*; heterogeneity in difference of *β_0 _*across comparisons					0.060	(0.002; 0.406)

**Figure 4:**
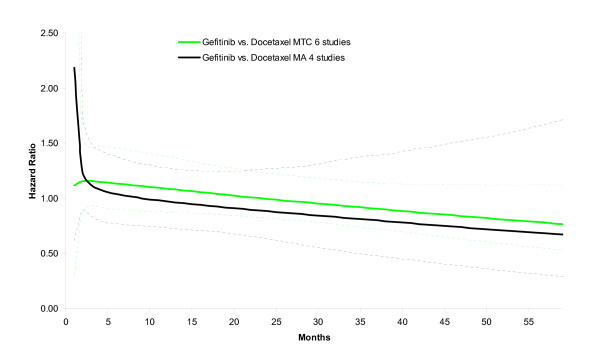
**Estimation of hazard ratio over time for gefitinib versus docetaxel as obtained with mixed treatment comparison model (4 gefitinib-docetaxel studies, 1 BSC-docetaxel study, 1 gefitinib-BSC study) is associated with less uncertainty than obtained with a meta-analysis model (4 Gefitinib-Docetaxel studies)**.

In the example there is both direct evidence (i.e. head-to-head studies) and indirect evidence (via BSC) for the comparison of gefitinib versus docetaxel. As such, the network meta-analysis combining both direct and indirect comparisons uses more information than a pairwise meta-analysis of the 4 gefitinib versus docetaxel studies. In Figure 4, the hazard ratio over time is presented for the pairwise meta-analysis of gefitinib versus docetaxel based on 4 studies, as well as the mixed treatment comparison. The estimates of the two analyses are comparable (at least from month 3 onwards) suggesting that inconsistency between direct and indirect estimates is not an issue of concern. However, the uncertainty of the hazard ratio over time is greater with the pairwise meta-analysis of 4 studies than the network meta-analysis of 6 studies. By incorporating indirect evidence the parameters of the fractional polynomial can be estimated more precisely in this example.

**Figure 5:**
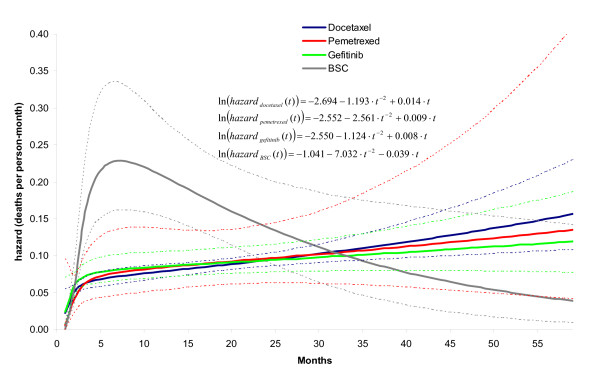
**Hazard over time for each of the interventions as obtained with random effects second order fractional polynomial (p1 = -2, p2 = 1) network meta-analysis model**. Docetaxel hazard curve used as 'anchor'.

**Figure 6:**
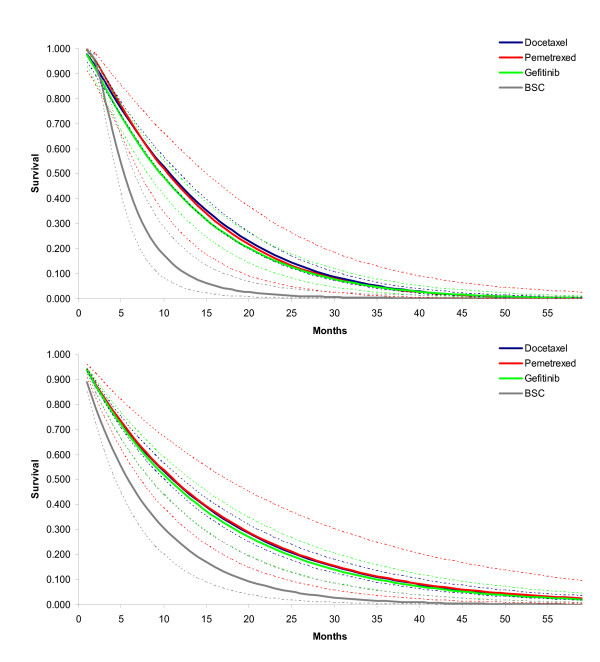
**Survival over time for each of the interventions as obtained with A) random effects second order fractional polynomial (p1 = -2, p2 = 1) network meta-analysis model; and B) random effects proportional hazards model assuming Weibull distribution**.

By using the average of study specific estimates for *β*_0_, *β*_1 _and *β*_2 _with docetaxel as the reference, the expected *β*_0_, *β*_1 _and *β*_2 _for the other interventions were calculated using the relative treatment effects *d*_0*Ak*_, *d*_1*Ak*_, and *d*_2*Ak*_. (See Table 4) The corresponding hazard and survival functions for each of the four interventions are presented in Figure 5 and 6A. With these parametric survival curves it is now possible to calculate the expected survival (i.e. the area under the curve) which is presented in Table 4 as well.

**Table 4:** Functions of parameter estimates for different fractional polynomials

	Fixed effects model, first order fractional polynomial		Fixed effects model, second order fractional polynomial		Random effects model (d_0_), second order fractional polynomial	
	**Median of posterior distribution**	**95% Credible Interval**	**Median of posterior distribution**	**95% Credible Interval**	**Median of posterior distribution**	**95% Credible Interval**

Docetaxel *						
β_0A _	-2.422	(-2.502; -2.348)	-2.689	(-2.882; -2.476)	-2.694	(-2.895; -2.513)
β_1A_	-2.174	(-2.988; -1.401)	-1.224	(-2.324; -0.198)	-1.193	(-2.267; -0.206)
β_2A_			0.013	(0.004; 0.022)	0.014	(0.006; 0.022)

Gefitinib						
β_0B _(β_0A _+*d_0AB_*)	-2.425	(-2.515; -2.325)	-2.531	(-2.776; -2.322)	-2.550	(-2.777; -2.273)
β_1B _(β_1A _+*d_1AB_*)	-1.540	(-2.527; -0.631)	-1.227	(-2.407; 0.082)	-1.124	(-2.433; 0.010)
β_2B _(β_2A _+*d_2AB_*)			0.007	(-0.002; 0.018)	0.008	(-0.002; 0.016)

BSC						
β _0C _(β_0A _+*d_0AC_*)	-1.652	(-1.818; -1.461)	-1.015	(-1.600; -0.480)	-1.041	(-1.554; -0.462)
β_1C _(β_1A _+*d_1AC_*)	-4.681	(-6.564; -3.010)	-7.082	(-9.677; -4.320)	-7.032	(-9.700; -4.622)
β_2C _(β_2A _+*d_2AC_*)			-0.040	(-0.071; -0.008)	-0.039	(-0.069; -0.010)

Pemetrexed						
β_0D _(β_0 A _+*d_0AD_*)	-2.402	(-2.616; -2.188)	-2.548	(-3.101; -1.954)	-2.552	(-3.093; -1.978)
β_1D _(β_1A _+*d_1AD_*)	-3.169	(-5.445; -0.673)	-2.583	(-5.634; 0.308)	-2.561	(-5.329; 0.167)
β_2D _(β_2A _+*d_2AD_*)			0.009	(-0.018; 0.033)	0.009	(-0.014; 0.028)

Expected survival (in months)						
docetaxel	12.5	(11.9; 13.3)	13.0	(12.2; 13.8)	13.0	(12.2; 13.9)
gefitinib	12.1	(11.3; 13.0)	12.2	(11.3; 13.2)	12.2	(10.6; 14.0)
BSC	7.2	(6.5; 8.1)	6.2	(5.1; 7.6)	6.2	(4.8; 7.9)
pemetrexed	12.7	(10.9; 14.7)	12.7	(10.5; 15.0)	12.9	(9.5; 17.4)

Difference in expected survival (in months)						
gefitinib vs docetaxel	-0.4	(-1.4; 0.6)	-0.8	(-1.9; 0.3)	-0.8	(-2.6; 1.1)
BSC vs docetaxel	-5.3	(-6.2; -4.3)	-6.8	(-8.0; -5.4)	-6.8	(-8.4; -5.0)
pemetrexed vs docetaxel	0.1	(-1.8; 2.2)	-0.3	(-2.5; 2.0)	-0.2	(-3.6; 4.4)
BSC vs gefitinib	-4.9	(-6.0; -3.8)	-6.0	(-7.4; -4.5)	-6.0	(-8.1; -3.9)
pemetrexed vs gefitinib	0.5	(-1.6; 2.8)	0.4	(-2.0; 3.0)	0.6	(-3.2; 5.3)
pemetrexed vs. BSC	5.4	(3.4; 7.6)	6.4	(3.9; 9.1)	6.7	(3.0; 11.4)

* (calculated as average from docetaxel study specific estimates: μ_0, _μ_1, _μ_2_)

**Figure 7:**
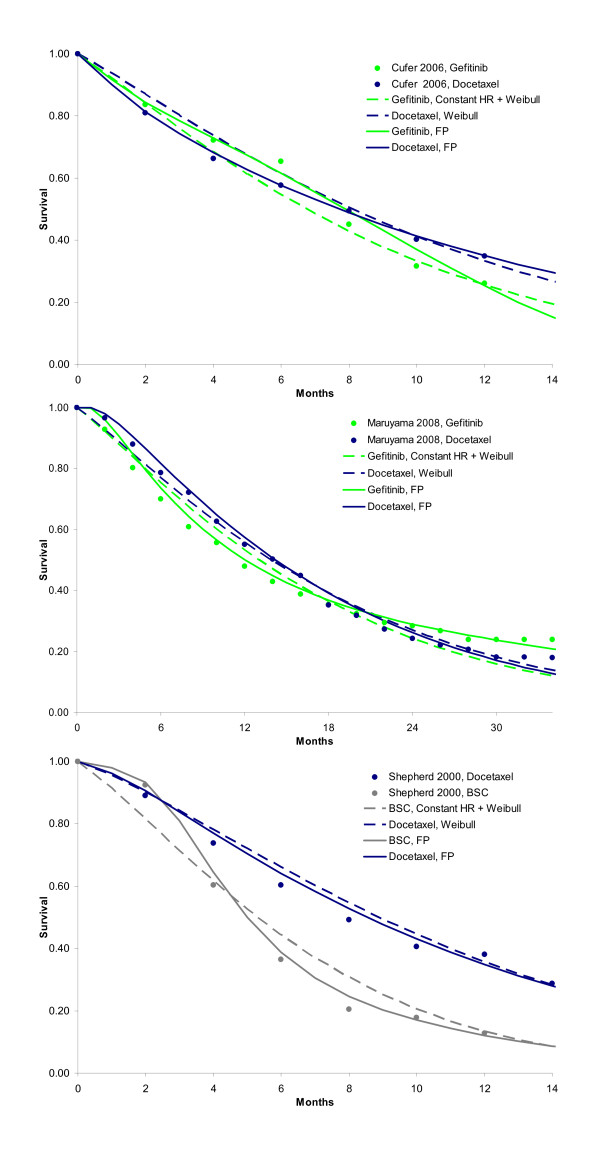
**Three representative studies that illustrate that a constant hazard ratio in combination with a Weibull reference curve does not fit the data as closely as the fractional polynomial models**.

When, as is common practice for cost-effectiveness analysis, a constant hazards ratio in combination with a Weibull distribution was assumed, the DIC of the model was 959.1. The fitted survival curves for docetaxel, gefitinib, BSC, and pemetrexed are presented in Figure 6B. The expected survival was respectively 15.1, 14.5, 8.0, and 15.2 months, and shows the overestimate relative to the random effects second order fractional polynomial model. The greatest difference is observed for the BSC survival curve, and the tails of the active interventions. To illustrate that the fractional polynomials produce a visibly better fit to the data than a simple model like the Weibull with a proportional hazards assumption, these models are presented for 3 studies in Figure 7. For the other 4 studies, the difference between the fractional polynomial curves and Weibull curves was not as great.

## Discussion

In this paper a method for (network) meta-analysis of survival data using a multi-dimensional treatment effect is presented as an alternative to synthesis of the constant hazards ratio. With first or second order fractional polynomials the hazard functions of the interventions compared in a trial are modeled and the difference in the parameters of these fractional polynomials within a trial are considered the multidimensional treatment effect and synthesized (and indirectly compared) across studies. In essence, with this approach the treatment effects are represented with multiple parameters rather than a single parameter or outcome.

Meta-analysis of survival data using the constant hazards ratio can be considered a special case of the model presented here. When in equation 6 *d*_1*Ak*_, *d*_2*Ak*_, ...*d_MAk _*equal 0, only the time independent parameters *β*_0*jk *_can be different across treatments within a trial and accordingly *d*_0*Ak *_reflect the constant log hazard ratio of treatment *k *relative to A. (Please note that the baseline hazard can still be modelled with multiple *β*_1*jk*_, *β*_2*jk*_, ..., *β_Mjk _*that can be different from 0, but these are constant across all interventions within a trial. With a Cox proportional hazards model the baseline hazard is unconstrained and not described by parametric distribution or function.) The advantage of the approach presented here is that it does not rely on the proportional hazards assumption and as a result the model used can be more closely fitted to available survival data. In a situation, where the violation of the proportional hazard ratio is less clear due to limitations of the data, it still can be considered useful modeling a multi-dimensional treatment effect to express the uncertainty in the violation of the assumption of proportional hazards.

For network meta-analysis it is important that for the relative effect measure of interest the transitivity assumption holds [3,12,13]. Although the transitivity assumption holds for the constant (log) hazards ratio, violations of the proportional hazards assumption within or across trials, can result in biased indirect and mixed treatment comparisons of relative survival over time. By incorporating additional parameters for the treatment effect, the proportional hazards assumption is relaxed and therefore indirect and mixed treatment comparisons are arguably less likely to result in biased indirect estimates.

With a (network) meta-analysis the value of randomization only holds within a trial, and not across trials [3,12,13]. In other words, patients are randomly assigned to treatments within a trial, but patients are not randomly assigned to different trials. As a result there is the risk that patients assigned to the different trials are not comparable. If the distribution of patient and study level characteristics that modify the relative treatment effects is not similar across trials indirectly compared results will be affected by confounding bias [13]. In the models presented in this paper, treatment effect estimates will be biased if there is an imbalance in the distribution of treatment*covariate interactions across studies regarding the multidimensional treatment effect. Hence, it is suggested to expand the current models by incorporating treatment*covariate interactions. An additional advantage is that it can explain heterogeneity and facilitates the prediction of expected survival for subgroups [13].

In the example analysis, aggregate level data, i.e. scanned Kaplan-Meier curves, were used for all interventions compared. However, the models can also be used in combination with individual patient level data, using a different likelihood. Patient-level analyses have the advantage that no (conservative) assumption has to be made regarding the censoring process. Furthermore, patient-level network meta-analyses have greater power to estimate meta-regression models thereby reducing inconsistency and providing the opportunity to explore differences in effect among subgroups. However, obtaining patient-level data for all RCTs in the network may be considered infeasible. As an alternative one could use patient-level data when available, and aggregate level data for studies in the network for which such data is not available thereby improving parameter estimation over aggregate-data-only models.

Drug coverage decision-making is often informed by cost-effectiveness analysis where expected costs and expected outcomes are compared. When the main objective of the competing interventions is to improve survival, the primary outcome of interest is expected survival or for-quality-of-life adjusted expected survival. Unfortunately, given the available follow-up in the clinical trials, survival data is often censored and therefore the expected survival cannot be obtained without extrapolation of the data over time. Standard practice is to extrapolate the available survival data for the reference treatment using a parametric survival function (e.g. Weibull, lognormal or log-logistic). This baseline hazard function is multiplied with the constant hazard ratio for each of the competing interventions relative to this baseline to obtain hazard functions for the interventions of interest. The assumption of a constant hazards function implies that only the scale of these parametric functions is affected by treatment, and accordingly all the competing interventions have the same shape. Since the tail of the survival function has a great impact on the expected survival this assumption may lead to biased or at least highly uncertain estimates regarding differences in expected survival and therefore cost-effectiveness estimates. Given the multi-dimensional treatment effect of the approach presented in this paper, the parametric hazards functions of the competing interventions can be different regarding all of their parameters. As a result the extrapolated survival functions for all the interventions are more closely fitted to the available data and expected survival is less likely to be over or underestimated. An additional advantage of the use of fractional polynomials is that models can be fitted that go to asymptotes, and are therefore far more stable at the ends than, say, standard polynomials or splines. Although the proposed models constitute a substantial liberalization for evidence synthesis of survival curves from RCTs, there is still a danger of under-stating the uncertainty in extrapolating the curves because the choice of fractional polynomials is based on model fit criteria. In order to reflect model uncertainty, it might be of interest to estimate the powers of the fractional polynomials as well.

## Conclusions

(Network) meta-analysis of survival data is commonly performed with models represented with one parameter for the relative treatment effect: the constant hazard ratio. When the proportional hazards assumption does not hold, models in which the treatment effect is represented by several parameters using fractional polynomials can be more closely fitted to the available data. The models allow straightforward estimation of expected survival to facilitate cost-effectiveness analysis.

## Abbreviations

AIC: Akaike information criterion; BSC: best-supportive care; DIC: deviance information criterion; NSCLC: non-small cell lung carcinoma; RCT: randomized controlled trial

## Competing interests

The author declares that they have no competing interests.

## Authors' contributions

JJ is responsible for the development of the concept and methods, analysis of the example and writing of the manuscript

## Appendix

### Extraction of data from survival curves to use in the network meta-analysis model

According to the Kaplan-Meier curve, the proportion of people alive at time point *t S_t _*that die between time point *t *and time point *t + *1 is equal to (*S_t _*- *S*_*t*+1_)/*S_t _*and can be described by binomial likelihood distribution: *r_t _*~ *bin*(*p_t_*, *n_t_*). Where is the number of deaths *r_t _*in the interval [*t,t+1*]. *n_t _*is the number of subjects at risk in that interval, and *p_t _*is the underlying risk.

In the absence of censoring for the interval [*t,t+1*], *n_t _*is the number at risk at the beginning of the interval and *r_t _*can be obtained by multiplying *n_t _*with (*S*_*t *_- *S*_*t*+1_)/*S*_*t*_.

The number at risk for a particular interval might be provided below the Kaplan-Meier graph; if not reported, it can be obtained according to  starting at the time point where *n_t _*is provided below the graph.

In the case of censoring, the overlap of the sequence of censoring and deaths within the time interval [*t,t+1*] is unclear, and it is not possible to derive the exact number of deaths and censoring in the interval. As extreme cases we can assume that, on the one hand, censoring occurs after the deaths within the interval, or, on the other hand, all censoring occurs before the deaths. In the first scenario *n_t _*is the number at risk at the beginning of the interval, whereas in the second scenario *n_t _*is the number at risk at the beginning of the interval minus the number of censored subjects. With the second scenario it is clear that *n_t _*and *r_t _*are smaller given (*S_t _*- *S*_*t*+1_)/*S_t _*resulting in more uncertainty regarding the estimate *p_t_*. To not underestimate the uncertainty we opted for the second scenario. Under the assumption that all censoring occurs before the deaths occur, *n_t _*can again be obtained by  with *n*_*t*+1 _reported below the graph, or based on the same calculation for the interval [*t+1, t+2*], etc.

### Winbugs code for second order fractional polynomial random effects network meta-analysis model

Model{

for (i in 1:N){         # N number of datapoints in dataset

# time is expressed in months and transformed according powers of fractional polynomial P1 and P2

time_transf1[i]<-(equals(P1,0)*log(time[i]) + (1-equals(P1,0))*pow(time[i],P1))

time_transf2[i]<-((1-equals(P2,P1))*(equals(P2,0)*log(time[i]) + (1-equals(P2,0))*pow(time[i],P2)) + equals(P2,P1)*(equals(P2,0)*log(time[i])*log(time[i]) + (1-equals(P2,0))*pow(time[i],P2) *log(time[i])))

# likelihood

# hazard over interval [t,t+dt] expressed as deaths per person-month

# r is deaths in interval, n is number at risk, h is hazard

r[i]~ dbin(p[i],n[i])

p[i]<-1-exp(-h[i]*dt) # cumulative hazard over interval [t,t+dt] expressed as deaths per person-month

# random effects model

# loop over datapoints

# s refers to study, k is intervention k, b is comparator

log(h[i])<-Beta[i,1]+ Beta[i,2]*time_transf1[i]+ Beta[i,3]* time_transf2[i]

Beta[i,1]<-mu[s[i],1]+delta[s[i],1]*(1-equals(k[i],b[i]))

Beta[i,2]<-mu[s[i],2]+delta[s[i],2]*(1-equals(k[i],b[i]))

Beta[i,3]<-mu[s[i],3]+delta[s[i],3]*(1-equals(k[i],b[i]))

}

# loop over studies

# NS is number of studies

# ks is intervention k, bs is comparator

for(m in 1:NS){

delta[m,1:3]~dmnorm(md[k,1:3],omega[1:3,1:3])

md[m,1]<-d[ks[m],1]-d[bs[m],1]

md[m,2]<-d[ks[m],2]-d[bs[m],2]

md[m,3]<-d[ks[m],3]-d[bs[m],3]

}

# priors

# NT is number of treatments

d[1,1]<-0

d[1,2]<-0

d[1,3]<-0

for(j in 2:NT){

d[j,1:3] ~ dmnorm(mean[1:3],prec2[,])

}

for(k in 1:NS){

mu[k,1:3] ~ dmnorm(mean[1:3],prec2[,])

}

omega[1:3, 1:3] ~ dwish(R[1:3,1:3],3)

# output SD and correlation based on estimated covariance matrix

sigma.theta[1:3,1:3] <- inverse(omega[1:3,1:3])

rho[1,2] <-sigma.theta[1,2]/sqrt(sigma.theta[1,1]*sigma.theta[2,2])

rho[1,3] <-sigma.theta[1,3]/sqrt(sigma.theta[1,1]*sigma.theta[3,3])

rho[3] <-sigma.theta[3]/sqrt(sigma.theta[2,2]*sigma.theta[3,3])

sd[1]<-sqrt(sigma.theta[1,1])

sd[2]<-sqrt(sigma.theta[2,2])

sd[3]<-sqrt(sigma.theta[3,3])

# output hazard ratio for month 1 to 60

# NT is number of treatments, c is reference treatment, k is treatment of interest, l is month

for (c in 1:(NT-1)) {

for (j in (c+1):NT) {

for (l in 1:60) {

t1[l]<-(equals(P1,0)*log(l) + (1-equals(P1,0))*pow(l,P1))

t2[l]<-((1-equals(P2,P1))*(equals(P2,0)*log(l) + (1-equals(P2,0))*pow(l,P2)) + equals(P2,P1)*(equals(P2,0)*log(l)*log(l) + (1-equals(P2,0))*pow(l,P2) *log(l)))

log(hazard_ratio[c,j,l])<-d[j,1]-d[c,1]+(d[j,2]-d[c,2])*t1[l]+(d[j,3]-d[c,3])*t2[l]

}}}

}

### WInbugs data structure

s[] study identifier

r[] incident cases in interval

n[] at risk at beginning of interval

k[] treatment

b[] comparator

time[] interval number/time point

s[]   r[]   n[]   k[]   b[]   time[]

1   7   81   2   1   1

1   5   74   2   1   2

1   7   69   2   1   3

1   6   62   2   1   4

.   .   .   .   .   .

.   .   .   .   .   .

.   .   .   .   .   .

7   53   134   3   1   3

7   70   160   3   1   4

7   12   90   3   1   5

7   22   78   3   1   6

END

# comparison by study (only used for random effects model)

ks[]   bs[]

2   1

3   2

2   1

2   1

4   1

2   1

3   1

END

## Pre-publication history

The pre-publication history for this paper can be accessed here:

http://www.biomedcentral.com/1471-2288/11/61/prepub
